# Remdesivir nephrotoxicity in tubular epithelial cells

**DOI:** 10.3389/fphar.2026.1876106

**Published:** 2026-09-03

**Authors:** Lucia Miño-Izquierdo, Juan Guerrero-Mauvecin, Natalia Villar-Gómez, Marta Hernández, Susana Carrasco, Rodrigo Castro-Gonzalez, Pablo Cannata, Maria D. Sanchez-Niño, Alberto Ortiz, Ana B. Sanz

**Affiliations:** 1 Laboratorio de Nefrología Experimental, Instituto de Investigación Sanitaria-Fundacion Jimenez Diaz (IIS-FJD), Universidad Autonoma de Madrid, Madrid, Spain; 2 RICORS2040, Madrid, Spain; 3 Department of Pathology, IIS-FJD, Universidad Autónoma of Madrid, Madrid, Spain; 4 Department of Pharmacology, Universidad Autonoma de Madrid, Madrid, Spain; 5 Instituto Reina Sofia de Investigación en Nefrologia, Madrid, Spain; 6 Department of Medicine, Universidad Autonoma de Madrid, Madrid, Spain

**Keywords:** acute kidney injury, antiviral drug, COVID-19, cytokine storm, nephrotoxicity

## Abstract

**Introduction:**

Remdesivir was the first antiviral drug approved to treat severe coronavirus disease 2019 (COVID-19). Nephrotoxicity was observed during preclinical development and longer duration of remdesivir therapy was associated with acute kidney injury (AKI) in patients with COVID-19, but its nephrotoxicity has been recently disputed.

**Methods:**

Remdesivir cytotoxicity was assessed in murine and human proximal tubular cells cultured in 2-D and 3-D models, as well as in a mouse model of acute kidney injury.

**Results:**

We now show that clinically relevant concentrations of remdesivir are cytotoxic to murine and human proximal tubular cells, either immortalized or in primary culture. Cytotoxicity was observed both in 2-D cultures and in spheroids and had morphological and functional features of necrosis. While mitochondrial dysfunction was observed, this appeared to be secondary to cytotoxicity and not a driver of cytotoxicity. Cell death could not be prevented by targeting proapoptotic caspases, nor by inhibiting necroptosis or ferroptosis programmed necrosis. Remdesivir metabolites were not cytotoxic. Interestingly, a short course of remdesivir that was not nephrotoxic by itself increased the severity of AKI induced by a cytokine storm elicited by bacterial lipopolysaccharide in mice. This is a clinically relevant context of use that may explain discrepancies related to the nephrotoxicity potential of short courses of remdesivir.

**Conclusion:**

Remdesivir induces necrotic tubular cell death resistant to current interventions targeting programmed cell death, that becomes clinically relevant in the presence of permissive kidney hyperinflammation.

## 1 Introduction

Remdesivir (GS-5734) was the first antiviral drug approved for treating severe coronavirus disease 2019 (COVID-19) and, more recently, for patients not requiring supplemental oxygen at an increased risk of severe disease (Food and Drug Administration, 2026a; European Medicines Agency, 2026a).

Remdesivir is rapidly converted within cells to its alanine metabolite GS-704277 and subsequently to nucleoside monophosphate, which is phosphorylated to yield the active nucleoside triphosphate GS-443902, which inhibits the RNA-dependent RNA polymerase of acute respiratory syndrome coronavirus 2 (SARS-CoV-2) and leads to delayed chain termination in the replication process (Gordon et al., 2022; Kokic et al., 2021). Alternatively, dephosphorylation of the nucleoside monophosphate generates the nucleoside (GS-441524), the predominant circulating metabolite, providing a potential source of active drug via a slow phosphorylation process. In healthy subjects, remdesivir and GS-704277 are primarily eliminated through metabolism while the nucleoside (GS-441524) is primarily excreted in urine (European Medicines Agency, 2026b). Plasma levels of remdesivir and its metabolites are higher in COVID-19 patients with impaired renal function (Sise et al., 2024; Sörgel et al., 2021).

In Europe, an estimated 100 million adults live with CKD (Vanholder et al., 2021). CKD is the comorbidity that most increase the risk of death from COVID-19 (Council and Group, 2021; Clark et al., 2020; Carriazo et al., 2022). CKD predisposes to acute kidney injury (AKI), as does COVID-19 and nephrotoxic medications, and AKI may accelerate CKD progression (Kidney Disease: Improving Global Outcomes KDIGO CKD Work Group, 2013; Rodríguez et al., 2018; Chawla et al., 2014; Liu et al., 2021; Hoste et al., 2015; Perazella and Rosner, 2022). Toxicology studies with remdesivir in rats and monkeys showed clear signs of renal damage and dysfunction. These were observable within 2 weeks in rats and less than 7 days in monkeys and, in most cases, were reversible after a 4-week recovery period (European Medicines Agency, 2026c). While regulatory authorities allowed its use in people with impaired kidney function (European Medicines Agency, 2026b), recent reports highlighted the recommendation of monitoring patients on remdesivir for AKI. In a retrospective cohort of COVID-19 patients prescribed remdesivir, 17% developed AKI (Alsowaida et al., 2025). Among 1103 adults with COVID-19, those with reduced kidney function had significantly lower odds of completing a full course of remdesivir (Lee et al., 2025). Furthermore, the cytotoxic effect of remdesivir was observed in primary tubular cells exposed for 3 days to clinically relevant concentrations (Sörgel et al., 2021; Xu et al., 2021). In addition, within shorter periods of time (24 h), remdesivir can inhibit tubular cell growth and impair their mitochondrial activity (Merches et al., 2022). We have now explored the molecular mechanisms of remdesivir nephrotoxicity in 2D cultures and in spheroids of human and murine proximal tubular cells as well as the interaction of remdesivir with cytokine storm syndrome in mice.

## 2 Methods

### 2.1 Cultured cells

MCT, a mouse kidney tubular cell line, was originally grown from SJL mice and was provided by Professor Eric G. Neilson of the University of Pennsylvania (Haverty et al., 1988). MCT were cultured in RPMI1640 (GIBCO, Grand Island, NY), supplemented with 10% FBS (fetal bovine serum, Sigma-Aldrich, Merck, Darmstadt, Germany), L-glutamine (2 mM concentration, Thermo Fisher Scientific, Waltham, MA), and streptomycin/penicillin (100 μg/mL and 100U/mL, Thermo Fisher Scientific). The human proximal tubular cell line HK2 (ATCC, Manassas, VA, United States), was cultured in RPMI1640 supplemented with 10% FBS, L-glutamine (2 mM), streptomycin (100 μg/mL), penicillin (100 U/mL), 36.23 μg/mL hydrocortisone and 1% Insulin Transfer Selenium (Thermo Fisher Scientific r). Primary Renal Proximal Tubule Epithelial Cells (RPTECs, ATCC) were cultured in REGM (Renal epithelial cell growth medium; LONZA, Basel, Switzerland). All cells were placed at 37 °C in 5% CO_2_.

Cells were seeded on the corresponding plates. After 24 h, they were changed to FBS-free medium and incubated an additional 24 h before stimulated. Stimuli (MedChemExpress, Monmouth Junction, NJ) were resuspended in DMSO at 1 mM (Remdesivir, GS-704277) and 10 mM (GS-441524) and used at concentrations between 0.5 and 10 μM, based on patient plasma data (3–12 μM) (Sörgel et al., 2021). The lethal cytokine cocktail TWEAK (100 ng/mL, Merck), TNFα (30 ng/mL, Peprotech, TX), INFγ (30U/mL, Peprotech) was used as a positive control of apoptosis induction (Justo et al., 2006). Mito-TEMPO (Santa Cruz) 10 μM, a specific mitochondrial antioxidant, and cell death inhibitors were added 1 h before remdesivir: the pan-caspase inhibitor zVAD 25 μM (Bachem, Bubendorf, Switzerland), the necroptosis inhibitor, Necrostatin-1 (Nec-1) 30 μM (Sigma-Aldrich), and the ferroptosis inhibitor (Fer-1) 1 μM (Sigma-Aldrich). Concentrations are based on previous experience (Martin-Sanchez et al., 2018).

### 2.2 Spheroid generation

Cells were seeded in round-bottom 96-well plates (Corning, Corning, NY) in 200 μL RPMI 1640 supplemented with 10% FBS. Each well contains 79 round-bottom microwells that enable the formation of spheroids of approximately 1,000 cells each. After 24 h, the medium was replaced with FBS-free RPMI, and cells were incubated for an additional 24 h prior to stimulation.

### 2.3 Cell viability

Cell viability in 2D cultures was assessed using the MTT assay. MTT is reduced by intracellular reductase enzymes in viable cells to insoluble formazan crystals. After 60 min of incubation, formazan was solubilized with DMSO and absorbance was measured at 570 nm using an Infinite 200 PRO reader microplate (TECAN, Männedorf, Switzerland). Cell viability in spheroids was determined using the Cell Counting Kit-8 (CCK-8, Sigma-Aldrich), based on the reduction of the tetrazolium salt WST-8 by cellular dehydrogenases in viable cells to a water-soluble formazan dye. Absorbance was measured at 450 nm 4 h after adding CCK-8. Absorbance correlates with the number of viable cells.

### 2.4 Cell cytotoxicity, cell death and cell cycle

Cell cytotoxicity was evaluated by measuring lactate dehydrogenase (LDH) release using the Cytotoxicity Detection Kit PLUS (Roche Science, Penzberg, Germany), according to the manufacturer’s instructions. Absorbance was recorded at 492 nm with 600 nm as reference using the microplate reader.

Cell death was assessed with Annexin V/7-AAD (BD Bioscience, Franklin Lakes, NJ) according to the manufacturer’s instructions. Cells were quantified by flow cytometry using a FACS Canto flow cytometer (BD Bioscience, Franklin Lakes, NJ), and data were analyzed with FACS Diva software (BD Bioscience). Early and late cell death was evaluated on PE fluorescence (Annexin V) versus PerCP (7-AAD) plots. Cells stained only with annexin V were considered early cell death; cells stained with both annexin V and 7-AAD were considered late cell death or necrosis.

To assess cell cycle, adherent cells were pooled with spontaneously detached cells and incubated in 100 μg/mL propidium iodide (PI), 0.05% NP-40, 10 μg/mL RNAse A in PBS at 4 °C for >1 h. This assay permeabilizes cells, thus PI stains both live and dead cells. The percentage of apoptotic cells (hypodiploid cells) and proliferative cells (phase S + G2/M) was counted by flow cytometry using FACS Canto cytometer and FACS Diva Software.

### 2.5 Morphology

For 3D spheroid morphological analysis, MCT and HK2 cells were seeded in micropatterned μ-Slide 8 Well high μ-Pattern ibiTreat plates (ibidi, Gewerbehof Gräfelfing, Germany). Spheroids were stimulated with remdesivir for 72 h and subsequently fixed with 4% paraformaldehyde and stained with DAPI (4′,6-diamidino-2-phenylindole; Sigma-Aldrich). Fluorescence was detected and photographed using confocal microscopy (Confocal System TCS SP5, Leica).

Cell morphology in 2D cultures was examined by transmission electron microscopy (TEM) in a Jeol Jem1010 (100Kv) microscope. Cells stimulated with remdesivir for 48 and 72 h were fixed in 4% formaldehyde/2% glutaraldehyde in PBS, dehydrated and embedded in Epon resin.

Nuclear morphology was assessed in formalin-fixed cells stained with DAPI (Sigma-Aldrich) and observed with fluorescence microscopy (Olympus BX53).

### 2.6 Mitochondrial membrane potential

MCT cells seeded in 12-well plates were incubated with TMRM 100 nM (Invitrogen) for 30 min at 37 °C in the dark. TMRM accumulates in mitochondria proportionally to mitochondrial membrane potential: mitochondrial depolarization decreases fluorescence intensity. Fluorescence was quantified by flow cytometry using a FACS Canto, and data were analyzed with FACSDiva software.

### 2.7 Clonogenic assays

MCT cells seeded in 12-well plates were stimulated with remdesivir for 24–72 h and then detached with trypsin-EDTA and reseeded in 100-mm dishes in RPMI1640 supplemented with 10% FBS and without remdesivir. After 72 h, cells were fixed with 4% paraformaldehyde, stained with crystal violet, and the dye was solubilized with methanol. Absorbance was measured at 570 nm using an Infinite 200 PRO microplate reader.

### 2.8 Intracellular ATP concentration

ATP levels were measured using the Luminescent ATP Detection Assay Kit (Abcam) in MCT cells following the manufacturer’s instructions and measuring luminescence in the Infinite 200 PRO reader microplate. ATP levels were subsequently normalized to total protein concentration determined using the bicinchoninic acid (BCA) assay (Thermo Fisher Scientific).

### 2.9 Animal model

Procedures were conducted in accordance with the NIH Guide for the Care and Use of Laboratory Animals after approval by the Animal Ethics Committees of IIS-FJD and Madrid Community (PROEX 56.5/23). C57BL/6 male mice (Charles River Laboratories, Ecully, France), 12- to 14-week-old, weighing 19–21 g were housed in cages at 25 °C ± 1 °C on a 12-h light/dark cycle and provided free access to food and water. Plasma samples were collected at the time of euthanasia. For euthanasian procedures, mice were anesthetized with ketamine 35 mg/kg (Ketolar; Pfizer, New York, NY) and xylazine 5 mg/kg (Rompun; Bayer, Leverkusen, GE) via intraperitoneal (i.p.) injection. Kidneys were perfused *in situ* with cold saline prior to removal. One kidney was snap-frozen in liquid nitrogen for RNA and protein studies. The remaining kidney was formalin-fixed and paraffin-embedded for histological studies. Plasma creatinine and BUN levels were measured using the enzymatic Creatinine Plus Version two reagent and the UREAL kit, respectively (Roche Diagnostics, Rotkreuz, Switzerland) on a Cobas Integra 400 analyzer (Roche Diagnostics).

Lipopolysaccharide (LPS)-induced endotoxemia is characterized by a cytokine storm syndrome (CSS) and interstitial leukocyte infiltration leading to kidney dysfunction and inflammation (Guerrero-Mauvecin et al., 2026). Mice (3-5 per experimental group) received an intraperitoneal injection of 5 mg/kg LPS (*Escherichia coli* O111:B4, Sigma-Aldrich-Merck), or vehicle (saline). The groups receiving remdesivir were given a loading dose of 40 mg/kg 2 hours after the LPS injection, followed by a maintenance dose of 20 mg/kg after 24 h. The mice were sacrificed 48 h after the LPS injection.

The dosing regimen for remdesivir was calculated based on the recommended adult human doses reported by the Spanish Agency of Medicines and Medical Devices (AEMPS), which include a 200 mg intravenous loading dose followed by a 100 mg daily maintenance dose. Human-to-mouse dose conversion factors were applied using allometric scaling based on the body surface area (BSA) (Nair and Jacob, 2016; Fontecha-Barriuso et al., 2020). Considering that drug administration in mice was performed intraperitoneally rather than intravenously, which may slightly reduce bioavailability, a loading dose of 40 mg/kg and a maintenance dose of 20 mg/kg were used.

### 2.10 TUNEL staining

TUNEL (deoxynucleotidyl-transferase-mediated dUTP nick-end labelling) staining was used to detect DNA fragmentation in dying cells. Kidney sections (3 μm) from paraffin-embedded samples were processed using the *In Situ* Cell Death Detection Kit (Roche) following the manufacturer’s instructions. Nuclei were counterstained with DAPI (Sigma-Aldrich). Slides were mounted with ProLong Gold Antifade Mountant (Thermo Fisher Scientific) and fluorescence was detected using an Olympus BX53 microscope. Positive nuclei were quantitated in 10 randomly chosen fields (400×) per kidney using Image Pro Plus Software (Media Cybernetics).

### 2.11 RNA extraction and real-time polymerase chain reaction

Tissue kidney samples were pulverized under liquid nitrogen. Total RNA was extracted using the TRItidy G reagent (Ref. A4051, PanReac AppliChem) and 1 µg was reverse transcribed with High-Capacity cDNA Archive Kit (Applied Biosystems, Foster City, CA). Quantitative PCR was performed in a Quant Studio 5 PCR System with the QuantStudio 3/5 Real-Time PCR Software using predeveloped primers (Applied Biosystems) and RNA expression of different genes was corrected for GAPDH expression.

### 2.12 Western blot

Whole frozen kidneys were pulverized under liquid nitrogen and lysed in T-PER Tissue Protein Extraction Reagent (Thermo Fisher Scientific) supplemented with 0.1% PMSF and 1% protease inhibitor (Sigma-Aldrich-Merck). Protein concentrations were determined using the BCA assay. Proteins were separated using Mini-PROTEAN TGX gels and transferred to nitrocellulose membranes (BIO-RAD). Membranes were blocked with 5% TBS/0.5% v/v Tween-20 skim milk and incubated overnight at 4 °C with the primary antibodies anti-NGAL (1:300, sc-515876, Santa Cruz Biotechnology, Santa Cruz, CA) and anti-KIM-1 (1:800, R&D Systems). After washing with TBS/Tween, membranes were incubated with secondary antibodies against mouse IgG (1:5000, GEHealthcare, Aylesbury, United Kingdom) for 1 h at room temperature. Detection was performed using ECL reagent (Thermo Fisher Scientific). Levels of expression were corrected for minor differences in loading using GAPDH (Millipore). Uncropped gel scans merged with molecular weight images for all presented western blots are shown in supplementary material (Supplementary Figure S1).

### 2.13 Immunohistochemistry

3 μm thick paraffin-embedded kidney tissue sections were stained using a PT-link device with a low pH solution. Sections were then washed with PBS for 5 min, blocked by incubation with 4% PBS/BSA + 10% serum and incubated overnight at 4 °C with primary antibody anti-NGAL (1:150, sc-515876, Santa Cruz Biotechnology) antibody. Sections were counterstained with Carazzi`s hematoxylin. NGAL staining was evaluated by a quantitative scoring system assessing stained kidney area, using Fiji software (ImageJ, version 1.54p) in 10 randomly selected fields (20x) per kidney. Data was expressed as % stained area. Samples were examined in a blind manner.

### 2.14 Statistics

Statistical analyses were performed using GraphPad Prism 8.02 (GraphPad Software, La Jolla, CA). Normal distributions were tested using the Shapiro-Wilk test prior to statistical analyses. Means for three or more groups were compared using ANOVA with Tukey’s *post hoc* test, one-way ANOVA for comparison with one independent variable and two-way ANOVA for comparison with two independent variables. Differences were considered statistically significant at p < 0.05. All experiments were performed at least three times.

## 3 Results

### 3.1 Cytotoxicity of remdesivir in cultured tubular cells

**FIGURE 1:**
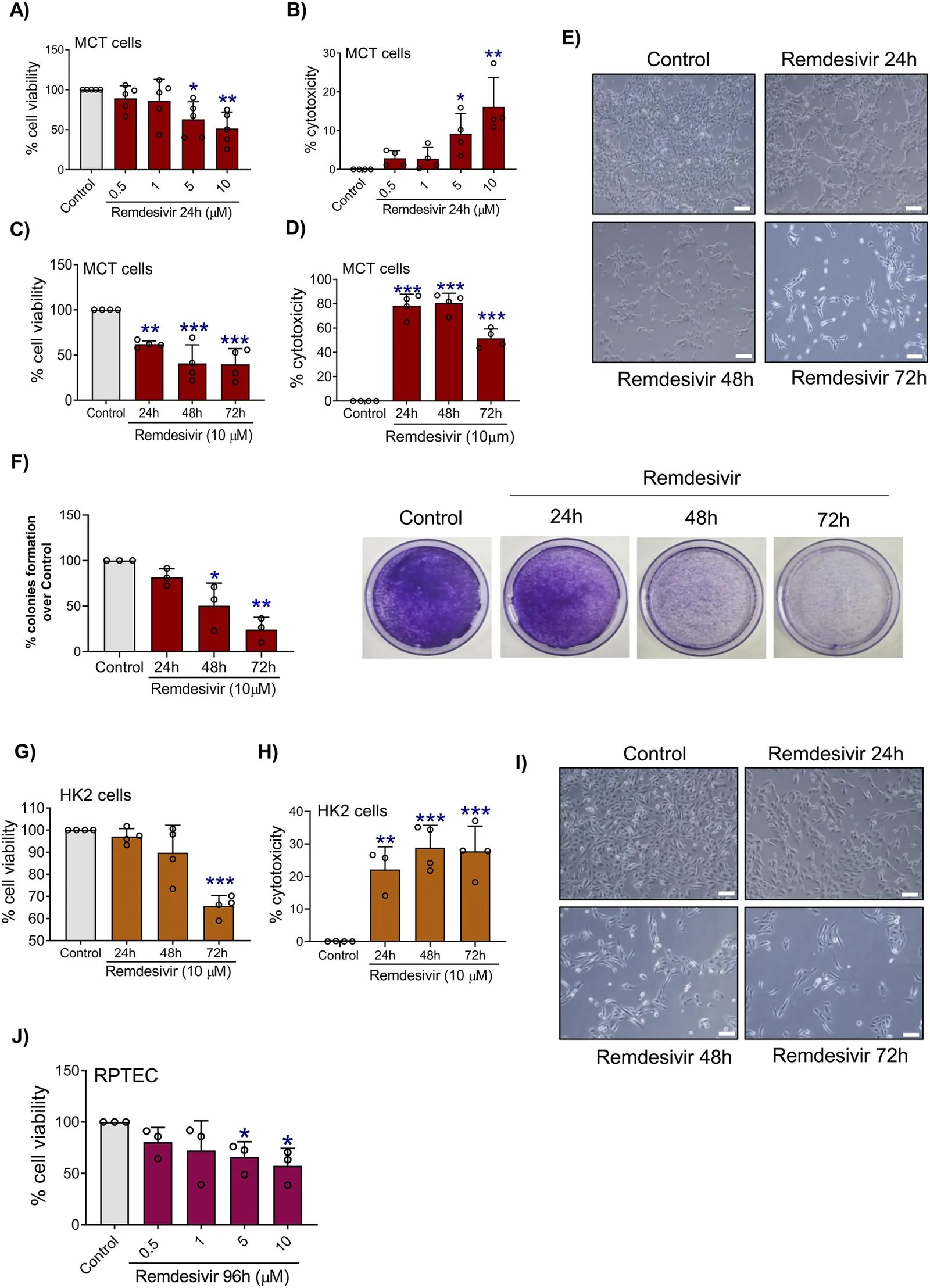
Cytotoxic effect of Remdesivir in cultured tubular cells. **(A,B)** The dose-response curve of remdesivir after 24 h on cell viability **(A)** and cytotoxicity **(B)** in the murine tubular cell line (MCT cells). **(C–E)** The changes in cell viability **(C)**, cytotoxicity **(D)** and cell detachment **(E)** induced by remdesivir at a concentration of 10 µM in MCT cells are time-dependent. **(F)** Exposure to remdesivir for 48 or 72 h induced irreversible damage in MCT cells, as no colonies formed 72 h after treatment while the controls did. **(G,H)** Effect of remdesivir at 10 µM on cell viability **(G)**, cytotoxicity **(H)** and cell detachment **(I)** in the line of human tubular cell HK2 is time-dependent. **(J)** Remdesivir also reduces cell viability in primary human tubular cells (RPTEC) at the same doses to those used for the tubular cell lines. **(A–J)** Mean ± SD of 3-5 independent experiments; *p < 0.05 vs. Control, **p < 0.01 vs. Control, ***p < 0.001 vs. Control. **(E,I)** Phase contrast images. Original magnification ×200 (scale 250 μm).

Remdesivir decreased murine MCT tubular cell viability as assessed by the MTT assay and increased cytotoxicity, assessed by LDH release kit (Figures 1A,B). Cell death induced by remdesivir was dose-dependent, peaking at 10 μM, the clinically relevant concentration observed in healthy volunteers (Sörgel et al., 2021; Humeniuk et al., 2020). Then, we performed a time-curve with 10 µM Remdesivir, observing that cell viability decreased and cytotoxicity increased at 48–72 h (Figures 1 C,D), coinciding with cell detachment (Figure 1E). In the colony formation (clonogenic) assay, prolonged exposure to the drug, for 48 and 72 h, inhibited colony formation, suggesting that extended treatment induces irreversible cellular damage (Figure 1F). Remdesivir also decreased cell viability, and induced cytotoxicity and cell detachment in immortalized human proximal tubular cells (HK2) (Figures 1G–I). Finally, remdesivir also reduced cell viability in primary cultures of human proximal tubular cells (RPTEC) (Figure 1J). Based on these results, we used a concentration of 10 µM to explore the molecular mechanisms of remdesivir-induced nephrotoxicity.

### 3.2 Cytotoxicity of remdesivir in 3D spheroids

**FIGURE 2:**
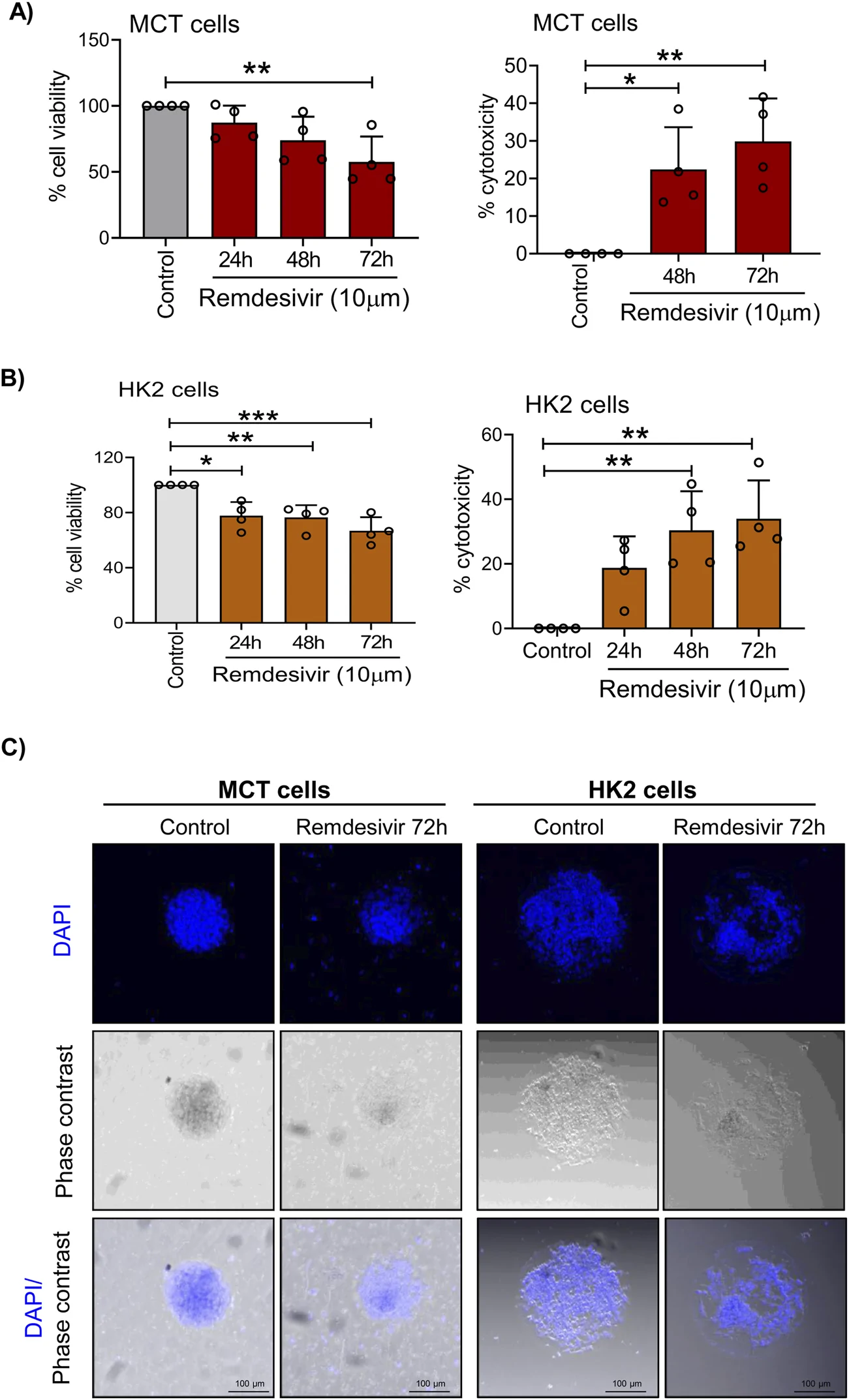
Cytotoxicity of remdesivir in 3D spheroids of tubular cells. **(A,B)** Cell viability and cytotoxicity of 3D spheroids of MCT cells **(A)** and HK2 cells **(B)** exposed to Remdesivir (10 µM) at different points of time. Mean ± SD of 4 independent experiments; *p < 0.05, **p < 0.01 vs., ***p < 0.001. **(C)** Nuclear DAPI staining and phase contrast of 3D spheroids from both MCTs and HK2 cells. Note that remdesivir treatment promotes the disaggregation of the 3D spheroids. Representative images are shown below. Confocal microscopy. Original magnification: ×200, zoom 4.

3D spheroids better represent the physiological spatial distribution of tubular cells. Remdesivir 10 µM also reduced cell viability and increased cytotoxicity in MCT spheroids (Figure 2A) resulting in disaggregation and loss of compactness (Figure 2C). Comparable results were observed in 3D HK2 spheroids (Figure 2B,C).

### 3.3 Remdesivir metabolites did not modify MCT cell viability

**FIGURE 3:**
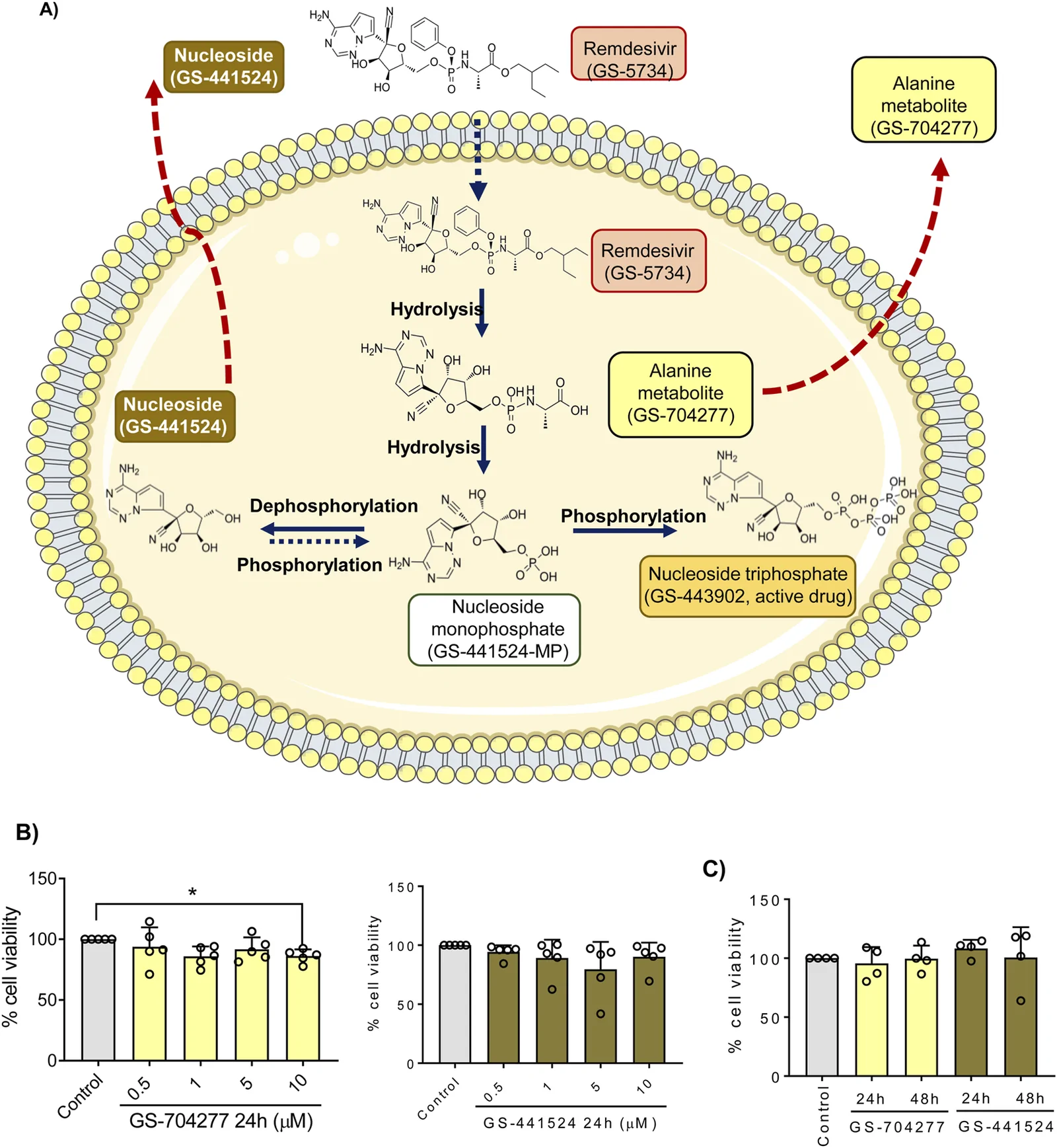
Remdesivir metabolites did not modify MCT cell viability. **(A)** Scheme of remdesivir metabolism. **(B)** The dose-response curve of the remdesivir metabolites found in plasma, GS-704277 **(A)** and GS-441524 **(B)** on MCT cell viability. Mean ± SD of 5 independent experiments; *p < 0.05. **(C)** GS-704277 and GS-441524 at 10 µM do not reduce MCT cell viability at longer times.

In healthy volunteers, remdesivir renal excretion ≈10% as unmodified drug and 50% as GS-441524 (European Medicines Agency, 2026b). Remdesivir is rapidly metabolized inside cells to the intermediate GS-704277, which is further converted into the active triphosphate (GS-443902). It also forms a metabolite (GS-441524) that can be slowly activated (Figure 3A). As GS-704277 and GS-441524 are detected in plasma, we evaluated their effect on MCT cells. Neither GS-704277 nor GS-441524 influenced cell viability at the same doses and times used for remdesivir (Figures 3B,C).

### 3.4 Characterization of remdesivir-induced tubular cell death

**FIGURE 4:**
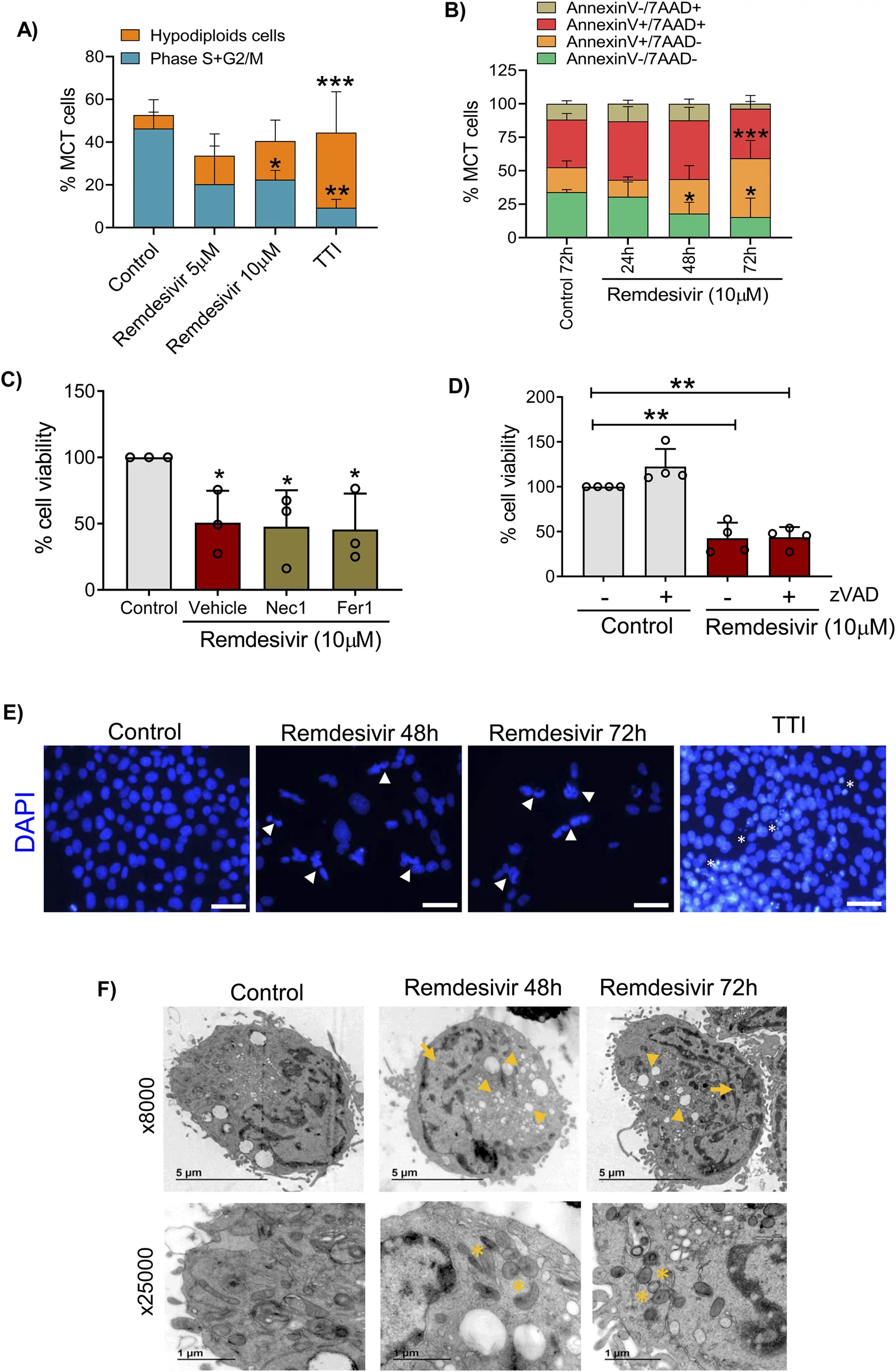
Characterization of Remdesivir-induced tubular cell death. **(A)** The percentage of hypodiploid cells (apoptotic cells) and cells in the S + G2/M phase (proliferating cells) was determined using flow cytometry to analyze DNA content 24 h after exposure to remdesivir. A TWEAK/TNFα/IFNγ (TTI) cytokine cocktail was used as a positive control for apoptosis. **(B)** MCT cells were exposed to remdesivir for the indicated periods of time, stained with annexin V/7-AAD, and cell death was analyzed by flow cytometry. **(C)** The pan-caspase inhibitor zVAD was added to MCT cells 1 hour prior to the addition of remdesivir, and cell viability was measured after 48 h. **(D)** Nec1 and Fer1 (necroptosis and ferroptosis inhibitors, respectively) were added to MCT cells 1 hour before remdesivir, and cell viability was measured after 48 h. **(A–D)** Mean ± SD of 3 independent experiments; *p < 0.05 vs. Control, **p < 0.01 vs. Control, ***p < 0.001 vs. Control. **(E)** DAPI-stained cells exposed to remdesivir (10 µM) for 48 and 72 h disclosed cells with irregular chromatin clumping suggestive of necrosis (arrowheads). TTI cytokine cocktail was used as a positive control for apoptosis. Fluorescence microscopy (x400). Note pyknotic, fragmented nuclei in the apoptosis control. Scale bars: 100 μm. **(F)** TEM of cells exposed to 10 μM remdesivir for 48 and 72 h disclosed cells with a typical necrotic morphology, characterized by nuclear swelling (arrow), partial cristae disruption (asterisk) and cytoplasmic vacuolization (arrowhead). Magnification ×8000 and detail x25000.

Next, we characterized the lethal effect of remdesivir on tubular cells. A previous study reported that remdesivir reduces tubular cell proliferation (Merches et al., 2022). Remdesivir weakly reduced the percentage of proliferating MCT cells and increased the percentage of hypodiploid cells at 24 h, typical of apoptosis. By contrast, the cytokine cocktail TWEAK/TNFα/IFNγ (TTI), a positive control of apoptosis induction, strongly increased the percentage of hypodiploid cells (Figure 4A). remdesivir increased AnnexinV positive cells, consistent with activation of programmed cell death (Figure 4B). However, remdesivir-induced cell death was not prevented by the pan-caspase inhibitor zVAD (Figure 4C), which inhibits apoptosis, nor by necroptosis or ferroptosis inhibitors (Nec-1, Fer-1) (Figure 4D). Additionally, cells treated with remdesivir showed irregular chromatin clumping typical of necrosis, while treatment with the cytokine cocktail TTI resulted in pyknotic and fragmented nuclei, characteristic of apoptosis (Figure 4E). Ultrastructural analysis by TEM revealed that remdesivir caused time-dependent ultrastructural damage. Control cells displayed preserved morphology and intact nuclei and mitochondria. Remdesivir led to nuclear swelling, partial cristae disruption and cytoplasmic vacuolization (Figure 4F). Overall, the results suggest that remdesivir-induced cell death exhibits features of necrosis that are independent of both necroptosis and ferroptosis.

### 3.5 Remdesivir-induced mitochondrial stress in tubular cells is not the cause of cell death

**FIGURE 5:**
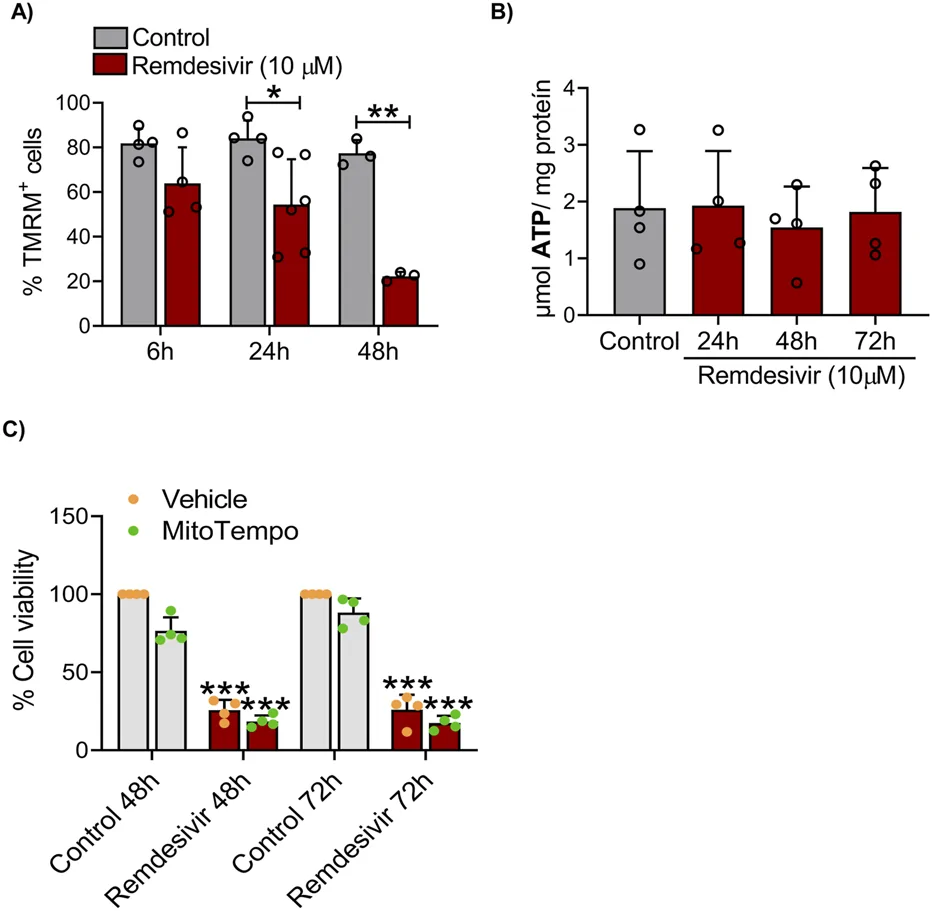
Remdesivir-induced mitochondrial stress in tubular cells. Tubular cells were exposed to remdesivir for the indicated periods of time. **(A)** Loss of mitochondrial membrane potential (MMP) was assessed by TMRM staining and flow cytometry. **(B)** ATP levels were assessed by a colorimetric assay and normalized by milligrams (mg) of protein. **(C)** MitoTempo (a scavenger of mitochondrial ROS) was added to MCT cells 1 hour before remdesivir, and cell viability was measured after 48 and 72 h. **(A–C)** Mean ± SD of 4 independent experiments; *p < 0.05 vs. Control, **p < 0.01 vs. Control, ***p < 0.001 vs. Control.

Tubular cells have a high energy demand due to their active transport mechanisms and mitochondria are crucial for their functions. Several drugs, including deferasirox, cisplatin, tenofovir and gentamicin, exert their nephrotoxicity in tubular cells through mitochondrial injury (Martin-Sanchez et al., 2017; Zhang et al., 2024; Hosseini et al., 2024; Upadhyay and Batuman, 2022). Remdesivir may affect mitochondrial function in cardiomyocytes and rat kidney NRK-52E epithelial cells (Merches et al., 2022). Remdesivir caused loss of mitochondrial membrane potential as assessed by TMRM staining (Figure 5A), but this did not translate into lower ATP levels when normalized for total protein (Figure 5B). In this regard, pretreatment of MitoTempo, a scavenger of mitochondrial ROS did not prevent remdesivir cell death (Figure 5C). These observations suggest that mitochondrial impairment may be a consequence, rather than a primary driver, of remdesivir-induced tubular cell death. Nevertheless, further studies will be necessary to exclude the potential role of early mitochondrial dysfunction in the initiation of this process.

### 3.6 Remdesivir increases the severity of cytokine storm syndrome associated AKI (CSS-AKI) in adult mice

**FIGURE 6:**
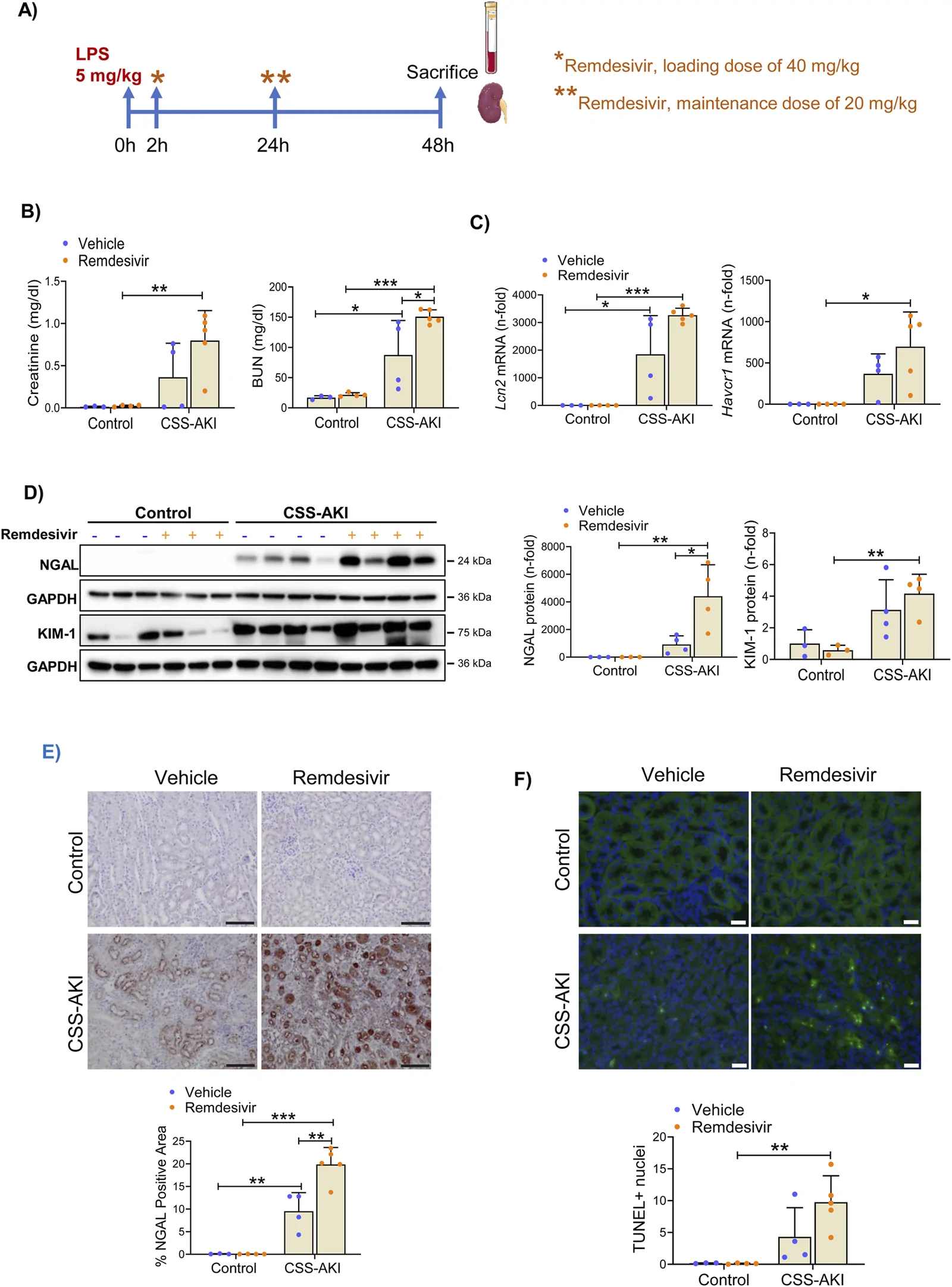
Remdesivir increases the severity of CSS-AKI in adult mice. **(A)** Mice were injected with a loading dose of remdesivir (40 mg/kg) 2 h after CSS-AKI induction and a maintenance dose of remdesivir (20 mg/kg) at 24 h. Mice were sacrificed 48 h after the CSS-AKI induction. **(B)** Serum creatinine and BUN levels. **(C)** Kidney mRNA expression of the renal injury marker *Lcn2* and *Havcr1*. **(D)** Kidney protein expression of the renal injury marker NGAL and KIM-1 encoded by *Lcn2* and *Havcr1* respectively. **(E)** NGAL staining and quantification. Original magnification, ×200. (Scale bars: 50 μm) **(F)** Cell death quantified by TUNEL staining. Representative images. Fluorescence microscopy. Original magnification ×400 (scale 50 μm). **(B–F)** Mean ± SD of 3–5 animals per group. *p < 0.05 vs. control; **p < 0.01 vs. control; ***p < 0.001 vs. control.

Finally, we explored the impact of remdesivir on a murine CSS-induced AKI (Figure 6A), given its context for use in patient with COVID-19 and CSS within a clinically relevant time frame (48 h). Remdesivir was not nephrotoxic within this time frame in healthy mice. However, it increased the severity of AKI in mice with CSS-AKI, as assessed by higher serum creatinine and BUN (Figure 6B) and higher levels of mRNA and proteins for tubular injury markers KIM-1 (encoded by *Havcr1*) and NGAL (encoded by *Lcn2*) (Figures 6C,D). Furthermore, NGAL overexpression, assessed by immunohistochemistry, and cell death, assessed by TUNEL staining, demonstrated that remdesivir-induced kidney injury in CSS-AKI mice was predominantly observed in the renal cortex (Figures 6E,F). These findings suggest that remdesivir may increase the severity of AKI induced by CSS.

## 4 Discussion

The key findings are that: a) clinically relevant remdesivir concentrations induce tubular cell death in 2D and 3D cultures, b) Remdesivir-induced cell death had features of necrosis (annexin V/7-AAD staining, LDH release, vacuolization and irregular chromatin condensation, weak presence of hypodiploid cells) and was not prevented by targeting well-characterized programmed cell death subroutines, c) an interaction with CSS-AKI was observed at clinically relevant remdesivir dosing: remdesivir increased the severity of CSS-AKI, a known complication of severe COVID-19, in mice. Unraveling the molecular and cellular mechanisms of remdesivir nephrotoxicity may allow the development of nephroprotective strategies.

Toxicology data in rhesus monkeys and rats showed that short remdesivir courses may cause severe nephrotoxicity. In male rhesus monkeys all doses tested (5–20 mg/kg/day) decreased renal function and promoted tubular injury in rats, doses >3 mg/kg/day for 4 weeks also caused kidney injury and dysfunction (European Medicines Agency, 2026b). However, clinical studies on remdesivir nephrotoxicity are controversial. Early pharmacovigilance studies showed an association between remdesivir and AKI incidence (Silva et al., 2021; Wu et al., 2022; Gérard et al., 2021). In clinical sobra la S trials, the incidence of AKI (“increased serum creatinine”) was 3-fold higher in patients treated for 10 days than in those treated for 5 days (15% vs. 5%) (Goldman et al., 2020; Food and Drug Administration, 2026b). However, in recent studies the current dose and timing of remdesivir administration were found safe, even in patients with an estimated glomerular filtration rate (eGFR) below 30 mL/min/1.73 m^2^ (Sise et al., 2024; Zhang et al., 2025; Umemura et al., 2025). Based on these studies, FDA and EMA approved in 2023 the use of remdesivir to treat COVID-19 in patients with severe renal impairment, including those on dialysis (Food and Drug Administration, 2026a; European Medicines Agency, 2026a). However, recent reports have emphasized the need to closely monitor patients receiving remdesivir for the development of AKI. The incidence of AKI in patients with COVID-19 treated with remdesivir was 17% (Alsowaida et al., 2025), and adults with reduced kidney function frequently do not complete full courses of remdesivir suggesting that impaired renal function may limit the completion of treatment, potentially due to safety concerns, clinical deterioration, or dosing considerations (Lee et al., 2025).

Preclinical studies are consistent with nephrotoxicity. Remdesivir reduced the number of immortalized rat kidney cells NRK-52E, without promoting cell death but was cytotoxic in RPTEC (Xu et al., 2021; Merches et al., 2022). This discrepancy may arise because immortalized cells acquire changes that can alter their response to stress, including exposure to drugs. We observed remdesivir cytotoxicity in both immortalized cell lines and primary cultures, suggesting that this effect is dependent on cell type. Furthermore, we observed remdesivir cytotoxicity in 3D culture models, and in mice with CSS-AKI, further supporting its potential cytotoxic effects on renal tubular cells. Remdesivir alone did not induce kidney impairment in healthy mice within the clinically relevant time-frame studied, but it increased the severity of CSS-AKI, which is consistent with its clinical context-of-use. These findings underscore the importance of tailoring remdesivir therapy to individual patients, particularly in those with pre-existing conditions such as renal impairment and severe COVID-19-related CSS.

The cellular mechanisms of remdesivir cytotoxicity are unclear. Mitochondria were suggested as targets. Remdesivir reduced the number of immortalized RPTEC/TERT1 cells, and impaired mitochondrial activity (Merches et al., 2022). However, in primary RPTEC cells, the mitochondrial changes induced by remdesivir appeared to be secondary to cytotoxicity rather than drivers of cytotoxicity (Xu et al., 2021). Consistently, in our studies, remdesivir lowered mitochondrial membrane potential, but ATP levels remained unchanged when adjusted for total protein content and MitoTempo did not restore cell viability. Similar findings were observed in cardiomyocytes, lung epithelial cells, and hepatocytes (Merches et al., 2022; DeFoor et al., 2023; Bjork and Wallace, 2021). Indeed, we characterized remdesivir-induced renal tubular cell death as consistent with necrosis. However, it did not appear to involve established necrotic pathways, including necroptosis or ferroptosis.

Remdesivir has a molecular weight of 602.6 g/mol. Cmax after a single dose of 200 mg has been reported to be 19.8 mg/L (32.9 µM) in hemodialysis patients and 7.8 mg/L in healthy controls (12.9 µM) (Sörgel et al., 2021). After a short course of an initial dose of 200 mg remdesivir followed by 5 daily doses of 100 mg, steady state Cmax was reported to be 2.16 mg/L (3.6 µM) in hospitalized patients with COVID-19 (European Medicines Agency, 2026b). Overall, the remdesivir concentrations tested in the present report can be considered clinically relevant and we observed a lethal effect on cultured renal tubular cells. In this regard, renal tubular cells exhibited the second-lowest CC_50_ for remdesivir (12.9 µM) in an *in vitro* cytotoxicity profile, consistent with the 10 µM dose used in our study (Xu et al., 2021).

The clinical formulation of remdesivir contains sulfobutylether cyclodextrin (SBECD), which is eliminated unchanged via the kidneys. In animal studies, accumulation of SBECD in plasma has been associated with liver and kidney toxicity (Luke et al., 2010). Because SBECD levels can also accumulate in patients with impaired renal function, it has been suggested that remdesivir-associated nephrotoxicity may be partly attributable to this excipient. However, no deleterious effects of SBECD on renal function have been observed (Luke et al., 2010; Shah et al., 2021). Our results further argue against SBECD mediating remdesivir nephrotoxicity as remdesivir alone was cytotoxic for cultured tubular cells and increased the severity of CSS-AKI.

Several limitations should be considered. Although a dose-response analysis was initially performed, further mechanistic studies relied on a single clinically relevant drug concentration in many experiments. The animal experiments had a relatively small sample size, following universal guidance on the performance of animal studies, which is enforced by the local ethics committee. Finally, we did not test an animal model of COVID-19 but relied on the LPS-induced cytokine storm model, which, unlike COVID-19, is sterile but reproduces some of the hyperinflammation features of severe COVID-19. Finally, we used previously healthy young mice, which may have underestimated the potential of remdesivir to cause kidney injury given that patients with severe COVID-19 frequently have predisposing factors. Among them, the most common are older age and CKD (Ortiz, 2024), (Clark et al., 2020), both of which are associated with low GFR, which increases exposure to remdesivir while additionally limiting kidney resilience (Marquez-Exposito et al., 2021). In conclusion, we have demonstrated that clinically relevant concentrations of remdesivir have direct cytotoxic effects on both murine and human tubular cells, under different culture conditions, and increased the severity of CSS-AKI, a context of use that reproduces some hyperinflammation features observed in COVID-19. The resulting tubular cell death exhibits characteristics of necrosis. Although the precise molecular mechanism driving this effect remains unclear, several targetable pathways were excluded. These observations may guide the design of clinical studies addressing remdesivir nephrotoxicity by demonstrating the *in vivo* requirement for a permissive environment consisting of kidney hyperinflammation. This requirement may have obscured the studies on the nephrotoxicity of the drug when the magnitude of hyperinflammation was not considered.

## Data Availability

The raw data supporting the conclusions of this article will be made available by the authors upon reasonable request to the corresponding author, in accordance with Frontiers' Publication Ethics and Data Availability guidelines and without undue reservation.
