# Supplementary material for: Remdesivir nephrotoxicity in tubular epithelial cells
Source: Front Pharmacol. 2026 Sep 3;17:1876106. doi: 10.3389/fphar.2026.1876106 (PMC13581931; doi:10.3389/fphar.2026.1876106)
Supplement: Supplementary file 1 [file Image1.pdf]

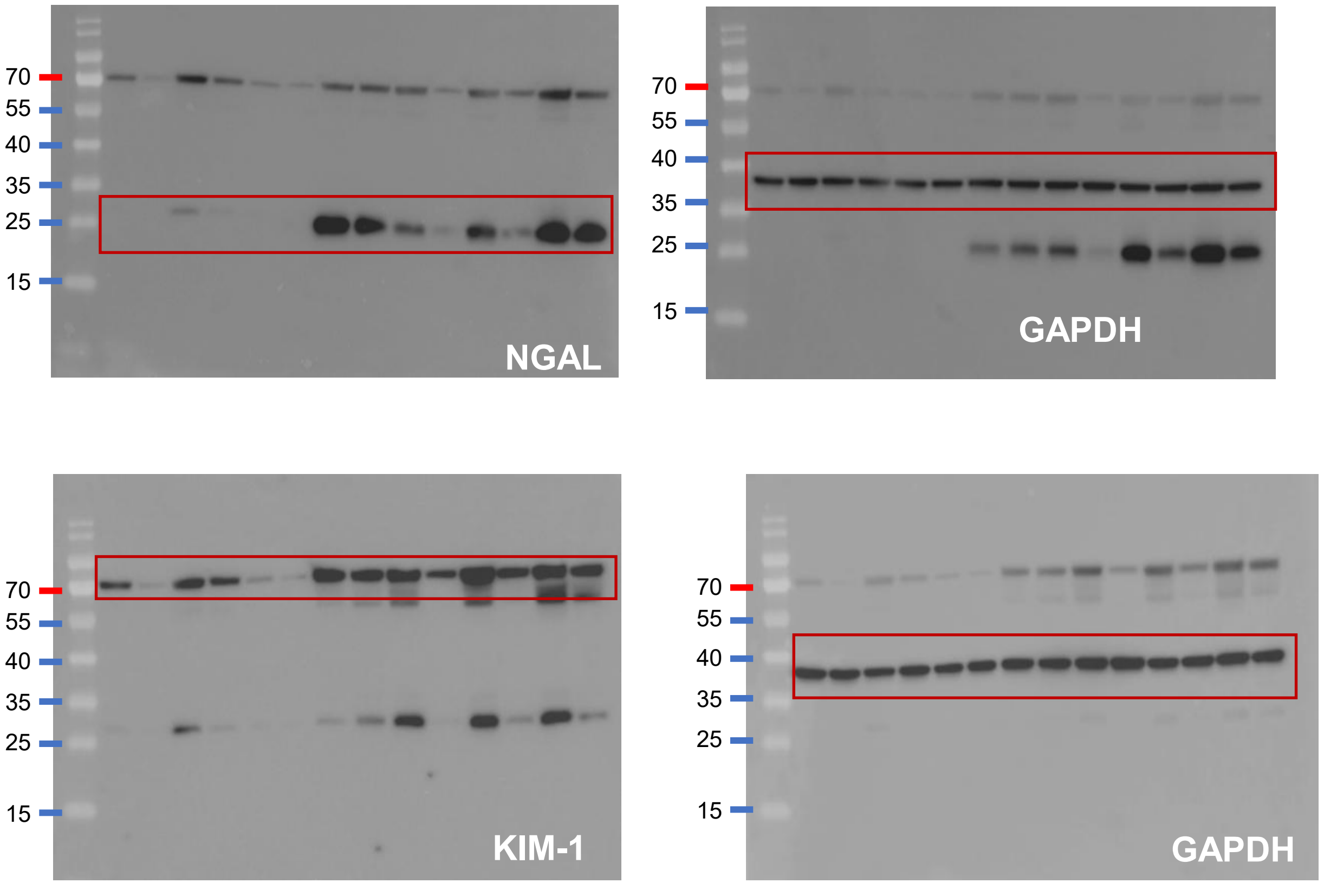

Figure S1. Uncropped gel scans merged with molecular weight images. Corresponds to presented western blot in figure 6.D
